# Human upper extremity motor cortex activity shows distinct oscillatory signatures for stereotyped arm and leg movements

**DOI:** 10.3389/fnhum.2023.1212963

**Published:** 2023-08-10

**Authors:** Clara Kwon Starkweather, Melanie A. Morrison, Maria Yaroshinsky, Kenneth Louie, Jannine Balakid, Kara Presbrey, Philip A. Starr, Doris D. Wang

**Affiliations:** ^1^Department of Neurological Surgery, University of California, San Francisco, San Francisco, CA, United States; ^2^Department of Radiology, University of California, San Francisco, San Francisco, CA, United States

**Keywords:** electrocorticography, stepping, arm swing, cortical oscillations, stereotyped movements

## Abstract

**Introduction:**

Stepping and arm swing are stereotyped movements that require coordination across multiple muscle groups. It is not known whether the encoding of these stereotyped movements in the human primary motor cortex is confined to the limbs’ respective somatotopy.

**Methods:**

We recorded subdural electrocorticography activities from the hand/arm area in the primary motor cortex of 6 subjects undergoing deep brain stimulation surgery for essential tremor and Parkinson’s disease who performed stepping (all patients) and arm swing (*n* = 3 patients) tasks.

**Results:**

We show stepping-related low frequency oscillations over the arm area. Furthermore, we show that this oscillatory activity is separable, both in frequency and spatial domains, from gamma band activity changes that occur during arm swing.

**Discussion:**

Our study contributes to the growing body of evidence that lower extremity movement may be more broadly represented in the motor cortex, and suggest that it may represent a way to coordinate stereotyped movements across the upper and lower extremities.

## Introduction

Stepping and arm swing are stereotyped, complex movements that integrate multiple coordinated muscle activations in the lower and upper limbs, respectively. The neural basis of these movements in humans is not well understood. For instance, it is not known whether correlates of stepping are confined to lower limb motor cortex and arm swing to upper limb motor cortex, or alternatively, if correlates of lower limb movements could be deciphered from upper limb motor cortical activity.

Motor cortical areas are thought to play a role in supraspinal control of stepping, both in trained stepping tasks in non-human primates and in human studies (Takakusaki, 2017). Lesion-based studies non-human primates trained to move in a bipedal fashion (Mori et al., 2006) revealed the necessity of motor and pre-motor cortex during stepping. Additionally, phases of the stepping cycle can be decoded from the primary leg motor cortical neural activity in monkeys during bipedal stepping (Fitzsimmons, 2009). In humans, there is a growing body of evidence that motor cortex plays a role in stepping as it occurs during natural gait. For instance, electroencephalography (EEG) recordings over the sensorimotor areas during walking show cyclical mu (8–13 Hz) and beta (14–40 Hz) band desynchronization throughout the gait cycle (Wieser et al., 2010; Gwin et al., 2011; Seeber et al., 2014, 2015). There is also coherence between beta band and leg muscle electromyogram (EMG) during the swing phase of walking (Petersen et al., 2012). Another recent work showed lateralizing alpha and beta band power fluctuations with initiation of contralateral limb walking movements (Nordin et al., 2020). Finally, phasic correlates of gait in the gamma band frequency have been captured using electrocorticography (ECoG) recordings over primary leg motor cortex (McCrimmon et al., 2018) during human treadmill walking.

These studies have focused on leg M1 activity during stepping and gait. One recent work highlighted heterogenous representation of multiple limbs in the hand knob area of M1 (Willett et al., 2020), and another study demonstrated regions associated with whole-body action planning throughout the classic M1 homunculus (Gordon et al., 2023), raising the possibility that motor correlates of stepping may be more broadly represented in the pre-central gyrus. Recently, by recording from chronically implanted electrodes from the subthalamic nucleus and the hand/arm M1 area in patients with Parkinson’s disease without gait disturbances, we found increased low frequency (4–8 Hz) subthalamic-M1 coherence time-locked to specific phases of the gait cycle (Louie et al., 2022). Based on these findings, we hypothesize that the hand/arm M1 area contains oscillatory correlates of stereotyped stepping.

One theory for lower frequency cortical oscillations during movement (Gwin et al., 2011; Petersen et al., 2012), is that oscillatory activity represents an efficient “top-down” means for controlling large groups of motor neurons (Baker et al., 1999; Fries, 2005). Specifically, low frequency oscillations (beta frequency or lower) are thought to modulate sub-threshold membrane potentials, creating coordinated windows of enhanced excitability that allow inputs to more easily trigger post-synaptic spikes (Fries, 2005). This sort of low frequency oscillation could be a useful coordinating mechanism for a movement such a stepping, which requires coordinated activation of multiple muscle groups across both sides of the body. In that case, we might expect to observe low frequency oscillations during stepping, even within the upper limb motor cortex, which could facilitate efficient synchronization of firing in downstream areas during automatic movements that integrate stepping, such as walking. In contrast, we might expect higher frequency (e.g., gamma) oscillations during arm swing, reflecting firing of upper limb motor cortical neurons. To test this hypothesis, we conducted recordings from a temporary subdural electrode strip placed over upper limb motor cortical areas in six subjects undergoing deep brain stimulation surgery for Parkinson’s disease or essential tremor. We recorded from a small number of subjects (*n* = 6 subjects) that performed visually cued and self-initiated stepping (*n* = 6 patients) or arm swing (*n* = 3 patients) tasks, while recording both biomechanical and electrophysiological data. In this limited dataset, we ask whether correlates of stepping exist in the hand/arm motor cortex, and whether these are separable from correlates of upper limb movement during arm swing.

## Materials and methods

### Subjects and electrode placement

**FIGURE 1:**
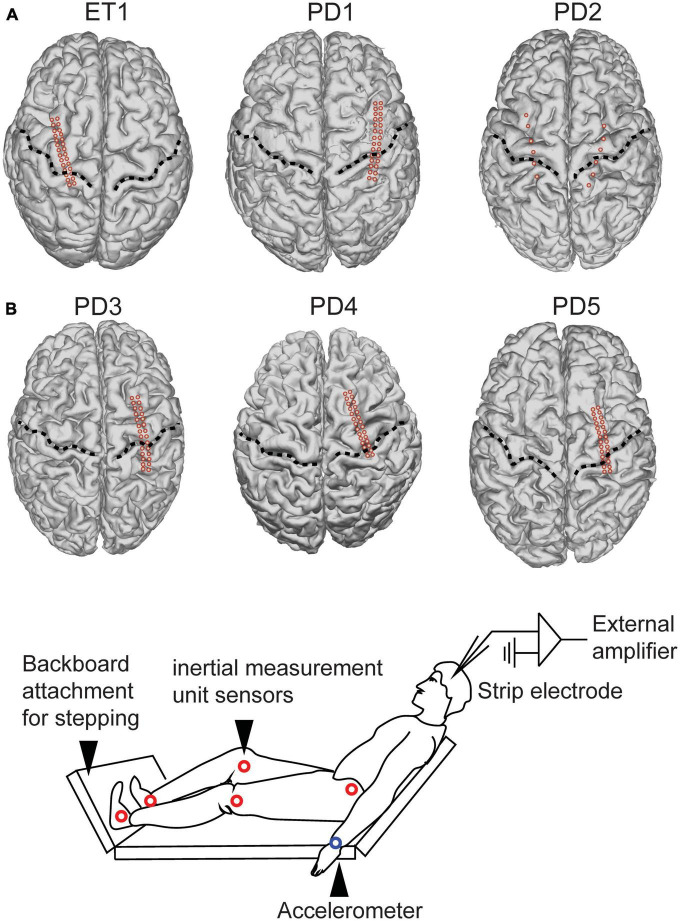
Subject electrode placement and task setup. **(A)** 1 essential tremor (“ET”) patient and 5 Parkinson’s disease (“PD”) patients underwent electrode strip placement approximately centered over upper limb primary motor cortex. Individual electrodes are designated by the red dots. Central sulcus designed by dotted line. **(B)** Intra-operative experimental setup. Subjects were positioned in a beach chair position with a backboard placed on the operating room bed to allow them to step onto a surface.

All patients provided written informed consent for participation in the study, approved by the institutional review board of the University of California, San Francisco (UCSF). Six subjects (2 female) included 5 subjects with idiopathic Parkinson’s disease (PD) and 1 subject with essential tremor (ET) (Figure 1A; Table 1). All subjects had clinical indications for Deep Brain Stimulation (DBS) surgery and were enrolled for the study at the University of California – San Francisco. Inclusion criteria for PD patients included baseline off-medication Movement Disorder Society – Unified Parkinson’s Disease Rating Scale Part III (MDS-UPDRSIII) scores between 30 and 80, greater than 30% improvement in MDS-UPDRSIII on-medication, and absence of significant cognitive impairment (>20 Montreal Cognitive Assessment). The ET patient had been diagnosed with 5 years of ET refractory to trial of multiple medications including a benzodiazepine. All patients provided written informed consent.

**TABLE 1:** Subject demographics.

ID	Age/Sex	Disease duration (years)	Disease state	UPDRS III gait sub-score	UPDRS III total score OFF meds/ TRS total score
ET1	47F	5	ET	n/a	27
PD1	63M	8	PD	1	24
PD2	54M	5	PD	1	25
PD3	72M	9	PD	1	29
PD4	76F	18	PD	1	37
PD5	75M	7	PD	1	40

UPDRS, Unified Parkinson’s Disease Rating Scale; TRS, tremor rating scales; PD, Parkinson’s disease; ET, essential tremor.

The mean age of PD subjects was 64.5 years, with standard deviation (SD) 10.9 years. The mean duration of disease for PD subjects was 9.4 years (SD 4.5 years). The age of the ET subject was 47 years, and her duration of disease was 5 years. MDS-UPDRS III gait disturbance subscore for all PD patients was 1, indicating mild gait disturbance.

All PD subjects underwent placement of unilateral or bilateral DBS leads into the subthalamic nucleus or globus pallidus interna. The ET patient underwent unilateral ventral intermediate nucleus (VIM) DBS implantation. Through the same burr hole used to implant the DBS lead, we placed a temporary 28-contact cortical paddle into the subdural space (Ad Tech, ref FG28A-MP04X-0T4) (Panov et al., 2017). The electrodes are placed just on the medial aspect of the hand knob area. To anatomically localize cortical paddle positioning, we fused postoperative CT images with preoperative MRI. The pre-operative T1 MRI was used to reconstruct cortical surface models in FreeSurfer (Fischl, 2012; Figure 1), and cortical contact projection onto a cortical surface mesh was performed using an open source pipeline.^1^

### Motor task and kinematic measurements

Subjects were positioned in a beach chair position with a backboard attached to the end of the bed to allow simulation of stepping on a hard surface (Figure 1B). Two systems were used for step or arm swing detection. Accelerometers were attached to the patient’s wrists and ankles (3-axis accelerometer) and Xsens MVN Analyze (Xsens Technologies, The Netherlands) was used. Accelerometry data was directly recorded using the external amplifier used for ECoG recordings. To synchronize the Xsens and neural recording, brief stimulation pulses were delivered at the onset of recording from the Xsens system. The Xsens system comprises 14 inertial measurement unit sensors placed over the patient’s body from the hip downward (Figure 1B). Subjects completed a visually cued arm swing and stepping task, in which they watched a video screen with a humanoid during walking or performing arm swing at a predetermined pace (1 gait cycle/second). They were asked to first mimic the stepping movements shown in a stepping video. They were next asked to mimic the arm swing movements shown in a separate arm swing video. The videos provided patients with guidance on how to perform the task. The video was then switched off prior to participants initiating their own movements during which the recordings reported here were conducted. All subjects completed a period of self-paced arm swing, as well as a period of self-paced stepping to simulate walking. All analyses reported in this manuscript are from the self-paced tasks unless otherwise specified. Gait and arm swing detection thresholds were performed in an automated fashion using threshold detection, and is reported in greater detail in Section “Results.”

**FIGURE 2:**
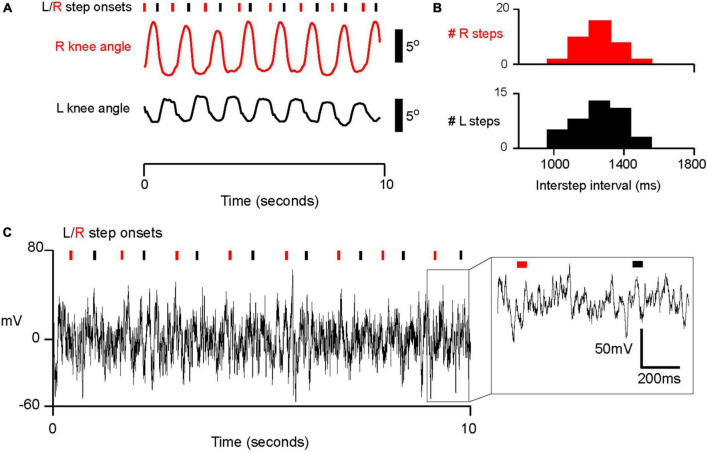
Example of intraoperative stepping data and electrophysiology in subject PD5. See analysis of accelerometry and inertia sensor offset in Supplementary Figure 1. **(A)** Example of semi-automated step detection for left and right steps. Inertial measurement sensors provided an estimate of knee angle. Right and left step onsets (denoted by red and black tracing and tick marks, respectively), were detected when the derivative of knee angle met a manually calibrated threshold. **(B)** Histogram of interstep interval (ISI) for left and right steps for PD05. M ± SD for R steps: 1.25 × 10^3^ ± 237 ms; for L steps: 1.29 × 10^3^ ± 281 ms. **(C)** Example trace of electrophysiological data from one electrode (electrode 6 in Figures 3, 4) in PD05 during walking.

Stepping events were detected by applying threshold detection to the derivative of knee joint angle. We detected events in a semi-automated fashion, and manually changed detection thresholds so that steps were reliably detected for each subject regardless of movement amplitude. We determined “step onset” as the time when the derivative of knee angle initially began to rise from a local minimum (see Figure 2A). In two subjects, Xsens system data were not available. For these two subjects (PD1 and PD2), we instead used ankle acceleration to detect step onsets. PD5 had both ankle accelerometry and joint angulation recorded, and his average inter-step interval (1.3 s) was similar to those of PD 1 and PD 2. We determined a relatively consistent offset between step onset detection by accelerometry and joint angulation in PD 3 (*M* = 179 ms, SD = 97.7 m, Supplementary Figure 1). Therefore, we relied on thresholding ankle accelerometry data and added an offset of 179 ms when determining step onsets for PD 1 and PD 2.

Arm swing events were detected by applying threshold detection to the accelerometers placed on the wrists. Similar to thresholding for step detection, we manually changed detection thresholds so that arm swings were reliably detected for each subject regardless of movement amplitude.

### Neural recordings, data processing, and data analysis

Local field potentials were recorded from subdural electrodes using the Neuro Omega system (Alpha Omega, Inc., Nazareth, Israel). A scalp needle electrode was used as a ground. Data were sampled at 22 KHz and high pass-filtered at 1 Hz. ECoG signals were processed and analyzed offline with MatLab (MathWorks, Inc., Natick, Massachusetts). We downsampled ECoG data to 1,000 hz, removed the DC offset, and applied a notch filter at 60 Hz and its harmonics. We performed common average referencing for each 1 × 14 row of electrodes on the 2 × 14 electrode paddle. In other words, we subtracted the average signal from electrodes 1–14 for the first 14 electrodes, and we subtracted the average signal from electrodes 15–28 for the second group of 15 electrodes. In these subjects, we performed an additional analysis to confirm that our results were robust to the choice of referencing. Specifically, we performed bipolar referencing in which the signal for each electrode was subtracted from the adjacent signal for each row of electrodes. For example, we referenced contact #2 to contact #1, and so forth. One subject (PD 1, see Figure 1A) had two 1 × 6 electrode paddles placed bilaterally. For this patient, bipolar referencing was performed on adjacent contacts on each paddle.

**FIGURE 3:**
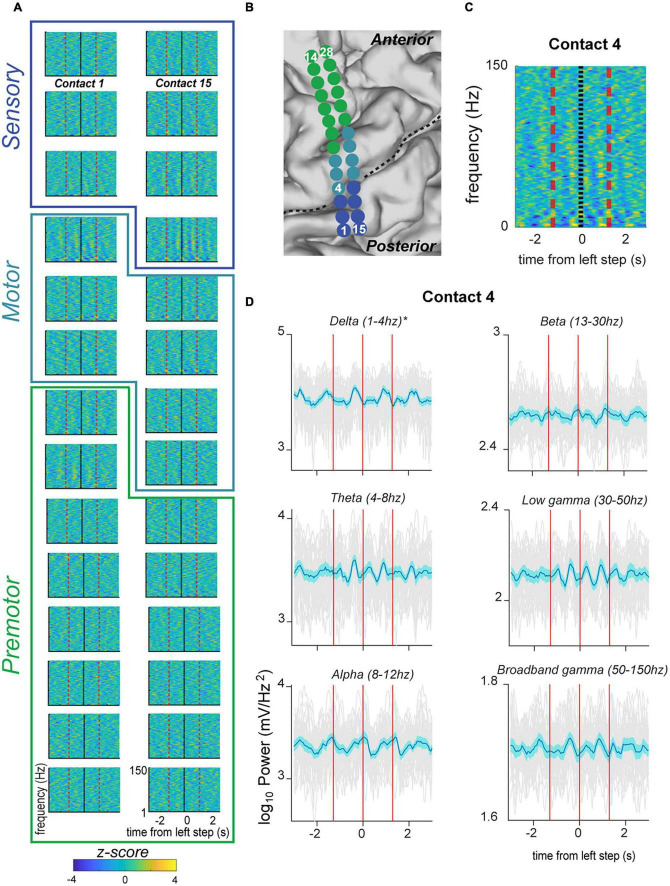
Fluctuation in signal power in various frequency ranges over the stepping cycle in electrodes located over the primary motor strip for PD05. **(A)** Spectrograms displaying power Z-scores for frequencies ranging up to 30 Hz. Black indicates left step onset (this subject had right sided electrodes) and dotted red line indicates inter-step interval (ISI). Colored outlines indicate correspondence onto anatomical regions (premotor, motor, sensorimotor) indicated in panel **(B)**. **(B)** Anatomical code for electrodes shown in panel **(A)** where green indicates, premotor, seafoam green indicates motor, and blue indicates sensorimotor cortex. Central sulcus designated with black dotted line. **(C)** Spectrogram for Contact 4 shown at higher magnification, and for frequencies ranging up to 150. This demonstrates the greater magnitude of power modulation in the lower frequency ranges. **(D)** Averaged power in canonical frequency bands, with ranges indicated in figure. Mean signal plus and minus two standard errors is plotted with the blue trace. Individual gray traces indicate individual trials.

For spectral analysis, we used the built-in MATLAB function “spectrogram” to return the short-time Fourier transform (STFT) of the signal. We used a fast Fourier transform of 512 points with 90% overlap to reduce edge effects. We computed the magnitude of power in various bands by taking the absolute value of the Fourier transform and averaging over pre-specified bands (delta, theta, alpha, beta, and gamma). All spectrograms comprise bootstrapped z-scores of magnitude of the Fourier transform. In order to do this, we calculated the standard error at each peri-event timepoint based on the amplitude of the Fourier transfer for each trial minus the average peri-event power over trials. We log 10 transformed all spectral power values over various frequency bands for peristimulus event plots (e.g., Figure 3C) and power values reported in the text.

### Statistical analysis

To determine whether frequency bands showed significant modulation over the stepping cycle, we first determined the timepoint at which the average absolute maximum power of a particular frequency band occurred within 1 inter-step (or inter-arm swing) interval of step onset. We determined the timing of maximum power (as opposed to power at an arbitrary pre-defined epoch relative to step onset) because cyclical power fluctuations contained maxima scattered throughout various timepoints relative to step onset (see Figure 3D for example). Using a paired *t*-test, we compared the power of each frequency band during a 500 ms interval containing the averaged maximum power amplitude, with 500 ms of time-shuffled data within the same interstep interval. Our method involved multiple comparisons (6 frequency bands for several motor electrodes per patient), so all significance values were Bonferroni corrected.

## Results

### Quantification and analysis of intraoperative stepping behavior

Six subjects were able to generate regular, cyclic stepping behavior in the operating room (Figure 1B). On average, the six subjects completed 33.2 steps with a standard deviation of 9.6 steps. All analyses were conducted on the averaged neural activity for all steps detected for each subject. We were able to detect cyclic changes in knee joint angle (Figure 2A) and ankle acceleration. Based on these, we measure movement initiation for each step, which we refer to as “step onset,” as shown for the example subject in Figure 2A. In addition, subjects’ step onsets occurred in a cyclical fashion at regular intervals which we term the inter-step interval (“ISI” for short). A histogram of ISI’s is shown for the same example subject in Figure 2B, with median ISI interval 1.29 s (SD 0.28 s). Chi square goodness of fit testing confirmed that the shape of each distribution of ISIs did not significantly deviate from a normal distribution (*p* > 0.05, Chi square goodness of fit test, all subjects). For all subjects, the median ISI’s ranged from 0.81 s to 2.38 s with standard deviations ranging from 0.081 s to 0.30 s for the longest and shortest median ISI, respectively. In summary, we showed that subjects took steps at regular intervals with a relatively narrow, grossly normal distribution of variation around median ISI, similar to those produced during gait.

### Motor cortical responses during stepping

We were able to obtain stable recordings, free of gross movement artifact related to stepping, while subjects performed the stepping task (Figure 2C). We recorded from ECoG electrodes placed over the premotor, motor, and sensory cortical areas (example subject in Figures 3A, B). Here we focus specifically on electrodes anatomically overlying the primary motor cortex (precentral gyrus) in the hand/arm area. Time-frequency analysis of electrophysiological data revealed phasic modulations in the amplitude of power spectra across multiple frequencies over a stepping cycle (Figure 3A). To measure the amplitude changes for individual canonical frequency bands, we averaged the log transformed power for delta (1–4 Hz), theta (4–8 Hz), alpha (8–12 Hz), beta (13–30 Hz), low gamma (30–50 Hz), and broadband gamma bands (50–150 Hz), which are plotted for the example subject PD5 shown in Figure 3C. For this subject, we found significant power modulation across the stepping cycle. For example, contact 4 located in the posterior aspect of upper limb motor cortex demonstrated delta power just prior to stepping that was significantly higher than shuffled baseline [see Section “Materials and methods–Statistics” log power of peak vs. baseline, median ± standard deviation: 3.21 ± 0.113 vs. 3.15 ± 0.0769, *t*(37) = 4.47, *p* = 3.99 × 10^–3^] (Figure 3C). Other frequency bands did not display significant modulation over the stepping cycle for this contact. These data are summarized in Figure 5A, where colors indicate contacts displaying significant modulation over the stepping cycle (*p* < 0.05, corrected for multiple comparisons). Specifically, stepping-related oscillatory modulation were seen in 2/7 contacts located over the motor cortex for PD5: contact 4 displayed significant modulation over delta frequencies, and contact 20 over the beta frequency band (Supplementary Figure 2A). In summary, this subject displayed significant modulation over predominantly lower frequency bands during stepping.

We next asked whether all 6 subjects demonstrated significant stepping-related modulation. We did qualitatively note that most modulation below the gamma-frequency range peaked in the half-ISI just prior to step onset (Supplementary Figures 2A, B). However, we performed our statistical analysis in an unbiased manner by asking if the average power in the 500 ms centered around the time of maximum power was significantly different than shuffled data (see Section “Materials and methods”), for each motor cortical contact in each subject. We found a similar result over all 6 subjects that performed the stepping task, including 1 essential tremor subject (“ET1” in Figure 5A). All subjects displayed significant modulation during stepping for at least one frequency band in the alpha range or lower. 13 out of a total 39 of motor strip contacts showed significant modulation during stepping. Of all contacts displaying significant modulation, 4/13 displayed significant modulation in the delta band; 7/13 in the theta band; 5/13 in the alpha band; 6/13 in the beta band; and 0/13 in either gamma frequency band (low or broadband). Some contacts displayed activation across overlapping low-frequency bands (meaning delta, theta, alpha, beta), including the anterior-medial contacts in ET1, PD2, and all contacts in PD1 (Figures 5A, B). In summary, stepping was associated with cyclical modulation in power amplitude in one third of contacts across multiple power spectra, limited to the delta, theta, alpha, and beta ranges, in motor cortical recordings centered over the upper limb motor cortex.

We next confirmed that our result was not dependent on our chosen method of common-average referencing in the 5/6 subjects in whom 2 × 14 electrode paddles were utilized (ET1, PD1, PD3, PD4, and PD5, Figure 1A). Analysis of data that was instead referenced using a bipolar scheme (see Section “Materials and methods”), showed that 4/5 of these subjects demonstrated significant modulation in low-frequency bands (delta, theta, alpha, beta). Specifically, 6 out of 27 bipolar-referenced contacts showed significant modulation during stepping (*p* < 0.05, corrected for multiple comparisons), whereas 0/27 contacts showed significant modulation in low- or high-gamma bands. Of all contacts displaying significant modulation, 2/6 demonstrated significant modulation in the delta band; 3/6 in the theta band, and 1/6 in the beta band.

One possibility is that the consistent low-frequency oscillations observed result from the propagation of higher frequency waves from premotor areas. We asked whether contacts located in the premotor cortex showed significant modulation during stepping. 3 out of 6 patients showed significant modulation over the stepping cycle (*p* < 0.05, corrected for multiple comparisons). Subject ET1 showed significant modulation in 3/10 electrodes in the delta frequency band. Subject PD2 showed significant modulation in 7/14 electrodes over the theta, alpha, beta, and both low- and high-gamma bands. Subject PD4 showed significant modulation in the alpha frequency band in 1/8 electrodes. The remaining 3 subjects did not demonstrate significant modulation over any frequency ranges. In summary, we did not report consistent oscillatory cortical activity during stepping over the premotor cortex in our dataset.

We next considered whether the low-frequency oscillatory activity observed during stepping may be related to movement artifact. Movement artifacts would be more likely to arise in channels with low signal-to-noise ratio. We measured signal to noise ratio by modeling the aperiodic component and periodic peaks in each motor cortical channel, and quantifying the power of peak periodic activity over aperiodic activity (Donoghue et al., 2020). We found that the peak power over aperiodic activity did not differ significantly between channels with significant stepping-related modulation [*p* = 0.97, *t*(387) = −0.0381]. We also asked if peak power over aperiodic activity differed significantly for motor cortical channels, within each subject, locked to movement initiation. For each subject, we computed peak power over aperiodic activity in the half ISI just prior to movement initiation compared to just following movement initiation and did not find significant differences [*p* = 0.45, *t*(131) = 0.76, for patient PD3, who exhibited the smallest *p*-value]. In summary, the low-frequency oscillatory activity observed during stepping cannot be easily explained by lower signal-to-noise in the channels displaying significant stepping-related modulation, or by movement-related changes in signal-to-noise ratio.

### Motor cortical responses during arm swing

**FIGURE 4:**
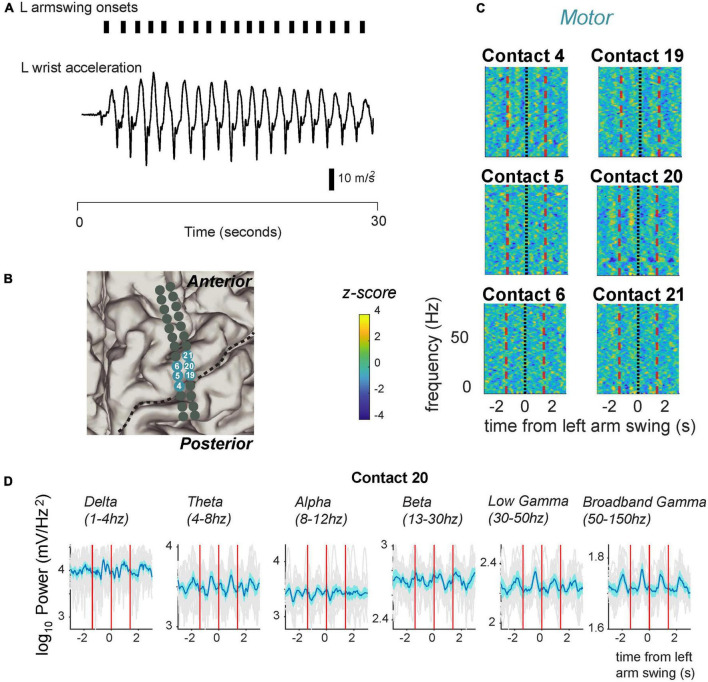
Fluctuation in signal power in various frequency ranges over the arm swing cycle in electrodes located over the primary motor strip for PD05. **(A)** Arm swing detection based on accelerometers attached to the patient’s wrist. We manually set thresholds to capture acceleration rising reliably over zero. **(B)** Anatomical code for electrodes shown in panel **(C)** where, seafoam green indicates motor cortex. Central sulcus designated with black dotted line. **(C)** Spectrograms displaying power Z-scores for frequencies ranging up to 30 Hz. **(D)** Averaged power in canonical frequency bands. Mean signal plus and minus two standard errors is plotted. Individual gray traces indicate individual trials.

We next asked whether motor cortical responses differed when subjects performed another stereotyped motor task involving upper limb movement. We had subjects perform arm swinging during the same intraoperative recording session, and recorded their movements with accelerometers attached to their wrists. Similar to stepping, we used wrist acceleration to detect movement onset (Figure 4A). We were able to obtain usable measurements in three subjects. On average, these three subjects completed 22 arm swing cycles with a standard deviation of 10.8 arm swings. All analyses were conducted on the averaged neural activity for all arm swings detected for each subject. We found that patients generated regular swinging movements with median inter-swing interval (ISwI) ranging from 1.38 s to 2.39 s with standard deviation ranging from 139 ms to 191 ms, respectively.

**FIGURE 5:**
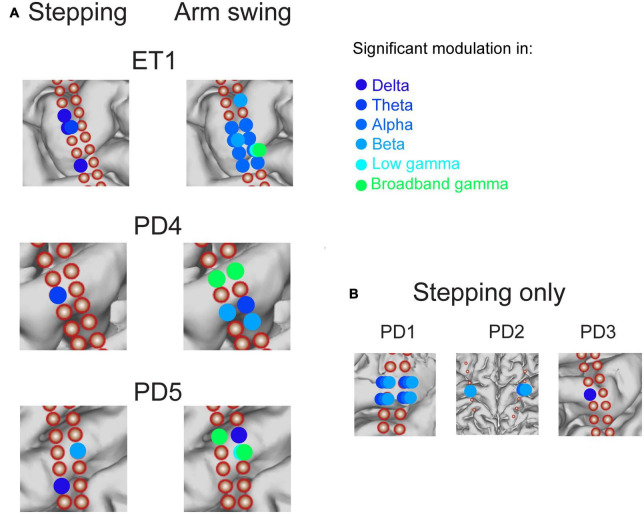
Summary of significant modulation across canonical frequency bands across all motor cortical electrodes in all subjects, during stepping and arm swing. Corresponding amplitude modulation across frequency bands indicated in Figure 6. **(A)** Comparison in 3 subjects in which stepping and arm swing data were available. Significant modulation in canonical frequency bands is indicated with colors ranging from dark blue to light green (see legend). A largely spatially distinct set of electrodes showed significant modulation in a set of frequency bands that included higher frequencies (low and broadband gamma) related to arm swing, compared to stepping. **(B)** 3 subjects in whom only stepping data were available. Consistent with the subjects shown in panel **(A)**, these subjects showed significant modulation in lower frequency bands spanning from delta to beta.

We demonstrate cyclical modulation over various frequency bands, over the ISwI, similar to stepping. However, these modulations tended to include higher frequencies spanning the gamma frequency range, and included a spatially distinct, partially overlapping set of electrodes. For example, the same example patient shown in Figure 3 (PD5, also see Figure 5A), demonstrated modulation in both the low and broadband gamma frequency range (Figures 4C, D, 5A) at contact 20. Low gamma (30–50 Hz) power frequency just prior to arm swing was significantly higher than shuffled baseline [log power of peak vs. baseline, median ± standard deviation: 2.29 ± 0.0537 vs. 2.25 ± 0.0534, *t*(16) = 4.38, *p* = 2.6 × 10^–3^] (Figures 4D, 5A). Broadband gamma (50–150 Hz) power was also significantly higher just prior to arm swing compared to baseline [1.86 ± 0.0411 vs. 1.82 ± 0.0244, *t*(16) = 5.50, *p* = 2.70 × 10^–3^] (Figures 4D, 5A).

**FIGURE 6:**
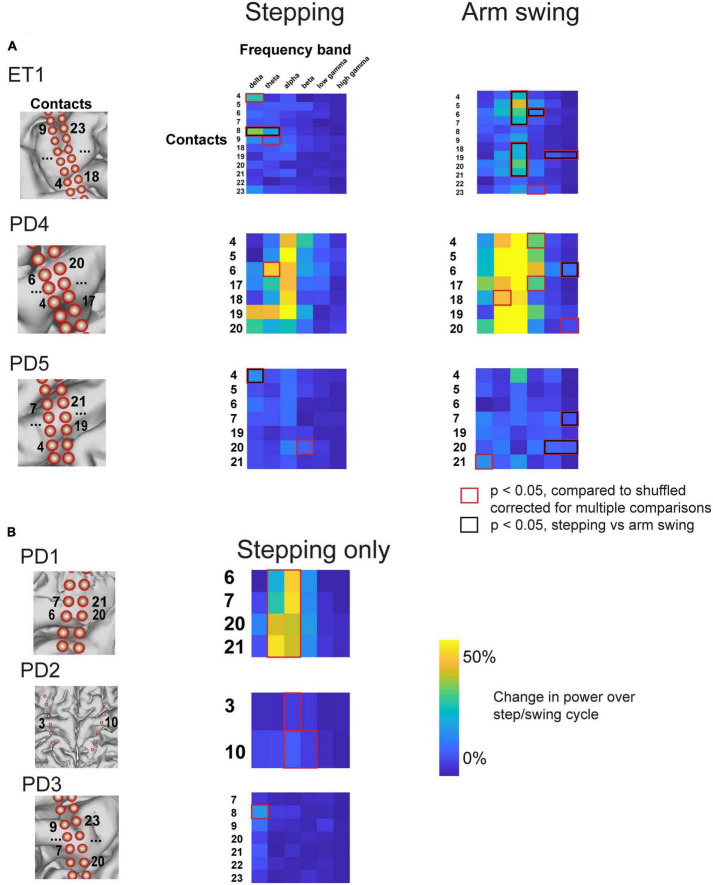
Percent change in amplitude during stepping and arm swing for all subjects. **(A)** Percent change in power over step/swing cycle, compared to shuffled baseline, for all subjects that completed both stepping and arm swing task. Those that accrued statistical significance, compared to shuffled baseline, are indicated with red boxes. Those contacts and frequency bands that also displayed statistically significant differences between stepping and arm swing are double marked with black boxes. Anatomical diagrams on the left hand indicate numerical codes for the contacts numbered on the heatmaps. **(B)** Percent change in power over step/swing cycle, compared to shuffled baseline, for all subjects that completed stepping task only.

We found a similar result over all 3 subjects that performed the arm swing task in addition to stepping (Figure 5A). All subjects displayed significant modulation over the ISwI in the broadband gamma frequency range in at least 1 contact, in contrast to no significant gamma modulation during stepping in the same 3 subjects. A total of 5/26 primary motor strip contacts showed significant broadband gamma modulation and 2/26 contacts showed significant low gamma modulation. Moreover, these contacts did not necessarily overlap spatially with those showing significant modulation during stepping, across all frequency ranges. For example, patient ET1 showed significant broadband gamma modulation in a relatively posterior electrode positioned over the hand knob during arm swing, but this same electrode did not show significant modulation during stepping (Figure 5A, Electrode 19 in Figure 6). In summary, we found significant modulation in the gamma frequency ranges during arm swing that was not present in these 3 subjects during stepping; significant modulation occurred in a spatially distinct set of contacts than those displaying significant modulation during stepping.

Similar to stepping, we also confirmed that our results were not dependent on common-average referencing scheme. Analysis of bipolar-referenced data showed that all three subjects demonstrated significant modulation during stepping in low- or high-gamma frequency bands. Specifically, 5/20 bipolar-referenced contacts showed significant modulation in the gamma frequency range (either low- or high-gamma, *p* < 0.05, corrected for multiple comparisons). 3/5 of these significant contacts demonstrated modulation in the low gamma range, and 2/5 of these significant contacts demonstrated modulation in the high gamma range.

To statistically validate our claim that arm swing corresponded to significant changes, particularly in higher frequencies, that were not present during stepping, we directly compared the magnitude of peri-event power modulation over shuffled data during stepping and arm-swing for all contacts showing significant modulation with either stepping or arm swing (two-sample *t*-test, degrees of freedom ranging from 22 to 63). This direct comparison shows that all 3 subjects that completed both arm swing and stepping displayed broadband gamma modulation in the arm swing peri-event period that was significantly greater than modulation see in the stepping peri-event period (Figure 6A, red boxes). We also found that the stepping-related modulation in the delta and theta range seen on electrode 8 for subject ET1, and the stepping-related delta modulation in the in the delta range seen on electrode 4 for PD5, were significantly higher than that observed during arm swing (Figure 6A, red boxes).

Lastly, we asked whether movement-related beta desynchronization was present during arm swing. We found that all three subjects that completed the arm swing task demonstrated significant beta frequency modulation in the half-ISwI before and after movement initiation (*p* < 0.05, corrected for multiple comparisons). Specifically, 10/26 motor cortical contacts demonstrated significant beta frequency modulation related to movement onset. Individual traces locked to movement onset for these motor cortical contacts showing significant beta frequency modulation are shown in Supplementary Figure 3. In contrast, no motor cortical contacts demonstrated significant beta frequency modulation related to movement onset in the half ISI before and after stepping. In summary, we identified expected movement-related beta synchronization during arm swing but not during stepping.

## Discussion

Here we report oscillatory signatures of stepping that differ from arm swing in ECoG recordings centered over the hand/arm motor cortex. We found neural oscillations during stepping in predominantly lower frequency ranges (delta, alpha, theta, beta), compared to those including the gamma frequency ranges for arm swing. Our results corroborate non-invasive recording studies utilizing scalp EEG, which have showed low-frequency modulations predominantly in the delta-to-alpha range during stepping (Presacco et al., 2011; Petersen et al., 2012; Seeber et al., 2014, 2015; Nordin et al., 2020).

### Role of motor cortex during gait and stepping

While is important to acknowledge that our recordings were conducted during a voluntary stepping task (as opposed to naturalistic walking), we provide some discussion as to how the phasic motor cortical activations during stepping could influence downstream motor activity during natural gait. As a complex behavior involving coordination of upper limb movements as well, we hypothesize that, based on our stepping data, walking may result in oscillatory changes in the motor cortical areas beyond the primary leg motor cortex.

Phasic modulations in canonical frequency band power during stepping has not been previously described using invasive intracranial recordings in humans. Prior studies demonstrating phasic fluctuations over sensorimotor cortex during walking have predominantly relied on non-invasive measures including EEG (Wieser et al., 2010; Gwin et al., 2011; Petersen et al., 2012; Seeber et al., 2014, 2015). These studies have described phasic signals predominantly spanning the beta (Petersen et al., 2012) to gamma (Seeber et al., 2015) frequency range over the course of a gait cycle. One recent study utilized subdural electrodes placed during along the leg motor cortex, and described electrophysiological changes during a variable speed walking task consistently across the gamma frequency band (McCrimmon et al., 2018). This study described a global increase in gamma frequency power during walking compared to resting, and also showed a significant correlation between walking speed and gamma band power. In other words, global properties such as time of gait onset and gait speed could be inferred from motor cortical activity, but phasic modulation of electrophysiological activity was not demonstrated on a single channel basis with each step. While our study does not focus on the leg motor cortex, it does broadly demonstrate significant modulation of motor cortical electrophysiology across the inter-step interval. Our data cannot address whether this phasic motor cortical activity influence gait on a step-by-step basis; however, our data argue that a precisely timed neural correlate of stereotyped stepping exists over the human primary motor cortex, which could serve to coordinate and entrain upper limb motor movements needed for a full body complex task such as walking. The phasic nature of the signals we describe is concordant with the phasic fluctuations in EEG signals captured over sensorimotor areas in the aforementioned works (Presacco et al., 2011; Petersen et al., 2012; Seeber et al., 2014, 2015; Nordin et al., 2020), which can increasingly provide some degree of spatial localization over motor areas. However, our result also localizes the motor strip, particularly upper limb motor strip, as a source of the prominent delta that carry significant decoding capability during gait.

Few prior studies have analyzed stereotyped activity during lower limb motor tasks. One prior study (Yin et al., 2022) analyzed dozens of seconds at a time of walking data in Parkinson’s disease patients, and described high gamma phase-amplitude coupling during walking trials in which the subject exhibit freezing. However, stereotyped oscillatory activities during individual steps had not been previously described over the upper limb motor cortex. Existing data on stepping has focused on the hindlimb motor cortex in invasive studies conducted on primates (see Fitzsimmons, 2009 for example) and in scalp EEG centered over the leg motor cortex in humans (Wieser et al., 2010; Gwin et al., 2011; Petersen et al., 2012; Seeber et al., 2014, 2015). Notably, our study demonstrated phasic modulation time-locked to the stepping cycle in upper limb motor strip and showed that this electrophysiological correlate of stepping is distinct in both spatial and time-frequency domains from neural correlates of a stereotyped upper limb task.

### Low frequency oscillatory activity during stepping

It is not known where low frequency oscillatory activity during stepping originates from. One possibility is that this activity propagates from gamma waves from the lower limb motor cortex. Another possibility is that this activity propagates directly from premotor cortex. We asked whether premotor cortical activity showed consistent significant modulation during stepping and found inconsistent results between subjects (see Section “Results,” “Motor cortical responses during stepping,” paragraph 4). However, this does not rule out the possibility that premotor activity drives the slow wave oscillations described in motor cortex. For instance, just a small surface area of premotor cortex was covered by our electrodes, making it difficult to definitively rule out consistent premotor activity cortical during the stepping cycle.

The presence of low frequency neural oscillations in the upper limb motor cortex during stepping raises the question of function. One hypothesis for the function of these low frequency oscillations draws from the “communication by coherence” hypothesis (Fries, 2005). In this hypothesis, low frequency oscillations generate fluctuations in sub-threshold membrane potentials, leading to excitability peaks that allow inputs to generate spikes with higher probability. Accordingly, there is a decrease in variability of neural spiking that coincides with the timing of beta oscillations (Murthy and Fetz, 1996). For example, Seeber et al. postulated that beta and mu (10–12 Hz) power changes in EEG centered over the lower limb motor cortex during walking related to state changes of cortical excitability (Seeber et al., 2014). In this way, low frequency oscillations could serve to entrain and coordinate groups of neurons to fire in a particular pattern. This could be useful in a task such as walking, which requires upper arm movements to occur in sync with lower limb movements. So, having a low frequency oscillation convey information pertaining to movement in the lower limbs could entrain groups of neurons to fire synchronously in the upper limb motor cortex during natural gait incorporating both upper and lower limb movement.

### Leg movement representation in the upper extremity motor cortex

It is well-known that the upper limb motor cortex contains a much more distributed representation than classically depicted within the homunculus (Schieber, 2001). For example, beta attenuation with upper limb reaching has been demonstrated in lower limb motor cortical area (Tobaa et al., 2016). Conversely, more recently, neural correlates of lower limb movements have been shown in upper limb motor cortex. Willett et al. showed that neurons in the upper limb motor cortex demonstrate changes in firing rate during ipsilateral and contralateral upper and lower limb movement (and intended movement) in quadriplegic patients (Willett et al., 2020). We did not consistently find significant broadband gamma activity, thought to represent spiking neural activity, during stepping. One possibility is that the recording technique used in our study [ECoG, as opposed to Utah arrays which could resolve individual spiking neurons in Willett et al. (2020)] was not sufficiently sensitive to detect frequency changes in the gamma range during stepping. For instance, there are obvious fluctuations in gamma amplitude over the stepping cycle for the subject shown in Figure 3C, but these did not accrue statistical significance due to a relatively small signal to noise ratio. Indeed, Willett et al. showed that contralateral arm movements were associated with the highest magnitude changes in neural firing rate over the hand knob, compared to contralateral leg, which could explain why changes in the broadband gamma range were detected for arm swing but not for stepping in our study.

### Limitations

Our study has several limitations. First, our task involved a stepping task performed in a supine position, rather than natural gait. Therefore, our results should be interpreted as pertaining to stereotyped lower limb movements rather than gait *per se*. Second, our study involved a relatively small sample size of six patients, of which three patients generated usable behavioral accelerometry to analyze in the arm-swinging task. This small sample size could bias our results. Third, we acknowledge that some authors argue low frequency power modulations may represented movement-related artifacts (Hell et al., 2018). However, we observe changes in spectral power during gait confined to a small range of frequency bands, whereas we would expect broadband changes related to movement artifact. In addition, we only observe fluctuations in particular low frequency bands across a distinct subset of electrodes based on walking versus arm swing. We would expect noise across all electrodes and movement types if our low frequency power changes were related to movement artifact.

Another important caveat is that our recordings were conducted in PD and ET patients. Therefore, brain activity over motor cortex may not reflect normal responses. One could argue that low frequency activity observed during stepping reflects modulations of pathologic beta band oscillations which are known to have pathologic elevation in Parkinson’s disease. These oscillations are suppressed by voluntary movement (Gillies et al., 2017; Tsiokos et al., 2017; Wang et al., 2018). One possible explanation for the low frequency oscillations observed during stepping in Parkinson’s patients, particularly in the beta band, is that these are simply modulations of pathologic beta band oscillations. However, we included one essential tremor patient that also exhibited significant modulation in the beta band during stepping, implying that the observed beta oscillations are not an artifact of Parkinson’s disease alone. Furthermore, we did not observe beta band or low frequency oscillations over all contacts, suggesting that the activity observed reflects localized somatotopic circuits rather than a global disease state. Finally, others have observed alpha and beta desynchronization upon voluntary movement initiation in both ET and PD patients, greater in magnitude than movement-related desynchronization observed in normal controls (Kondylis et al., 2016). Importantly, this difference between ET and PD patients and normal subjects was not observed in delta and theta frequency bands, which we observed in 5 of 6 subjects during lower limb movements (Figure 5). So, the lowest frequency oscillations below 8 Hz described in our results cannot be explained by previously described pathologic oscillations in ET and PD. Furthermore, there were no intra-group differences reported in gamma frequency oscillations, which we observed during upper limb movements. Therefore, the broadband gamma signals reported in our results are unlikely to result from pathological oscillations.

## Conclusion

Our study is the first to compare electrophysiological correlates of stereotyped upper and lower limb movements in the upper limb motor cortex. We showed that upper limb motor cortex shows phasic activation in distinct frequency bands during stereotyped upper and lower limb movements. In line with prior non-invasive EEG studies, we show that low frequency oscillations correlate with lower limb movements. In particular, power modulation over canonical lower frequency bands during stepping could serve an important role in synchronizing movement in other parts of the body, such as arm swing during gait. These low frequency modulations could serve an important role in coordinating natural gait. Our study showcases the need for further studies to investigate the underlying mechanisms of the distinct oscillatory signatures of motor cortical activity during stereotyped movements.

## Data availability statement

The raw data supporting the conclusions of this article will be made available by the authors, without undue reservation.

## Ethics statement

The studies involving humans were approved by the institutional review board of the University of California, San Francisco. The studies were conducted in accordance with the local legislation and institutional requirements. The participants provided their written informed consent to participate in this study.

## Author contributions

CS analyzed data, generated figures, and wrote the manuscript. DW designed the experiments and wrote the manuscript. MM analyzed imaging data and generated a figure. MY, KL, JB, KP, DW, and PS collected the data. All authors contributed to the article and approved the submitted version.

## Footnotes

1
https://edden-gerber.github.io/ecog_recon/

## Supplementary material

Supplementary Figure 1 Analysis of offset between step detection by accelerometry and step detection by inertia sensor in PD05. **(A)** Trace of ankle acceleration and knee angle in PD05, demonstrating noisier trace for accelerometer measurements but consistent timing and offset of acceleration trace and inertia sensor trace. **(B)** Histogram of offset between the two methods of detection. *M* = 179 ms ± SD = 97.7 ms.

Supplementary Figure 2 Traces for all motor cortical contacts illustrating normalized power during stepping and arm swing for all subjects. **(A)** Traces of normalized power in 3–12 Hz, 14–40 Hz, and 50–150 Hz frequency ranges for subjects in which both arm swing and stepping were recorded. Each trace is one contact. **(B)** Traces of normalized power in the same frequency ranges for subjects in which stepping only was analyzed. Each trace is one contact.

Supplementary Figure 3 Traces for all motor cortical contacts demonstrating significant beta modulation with movement initiation during arm swing.
