# Supplementary figures and images for: Human upper extremity motor cortex activity shows distinct oscillatory signatures for stereotyped arm and leg movements
Source: Front Hum Neurosci. 2023 Aug 10;17:1212963. doi: 10.3389/fnhum.2023.1212963 (PMC10449648; doi:10.3389/fnhum.2023.1212963)

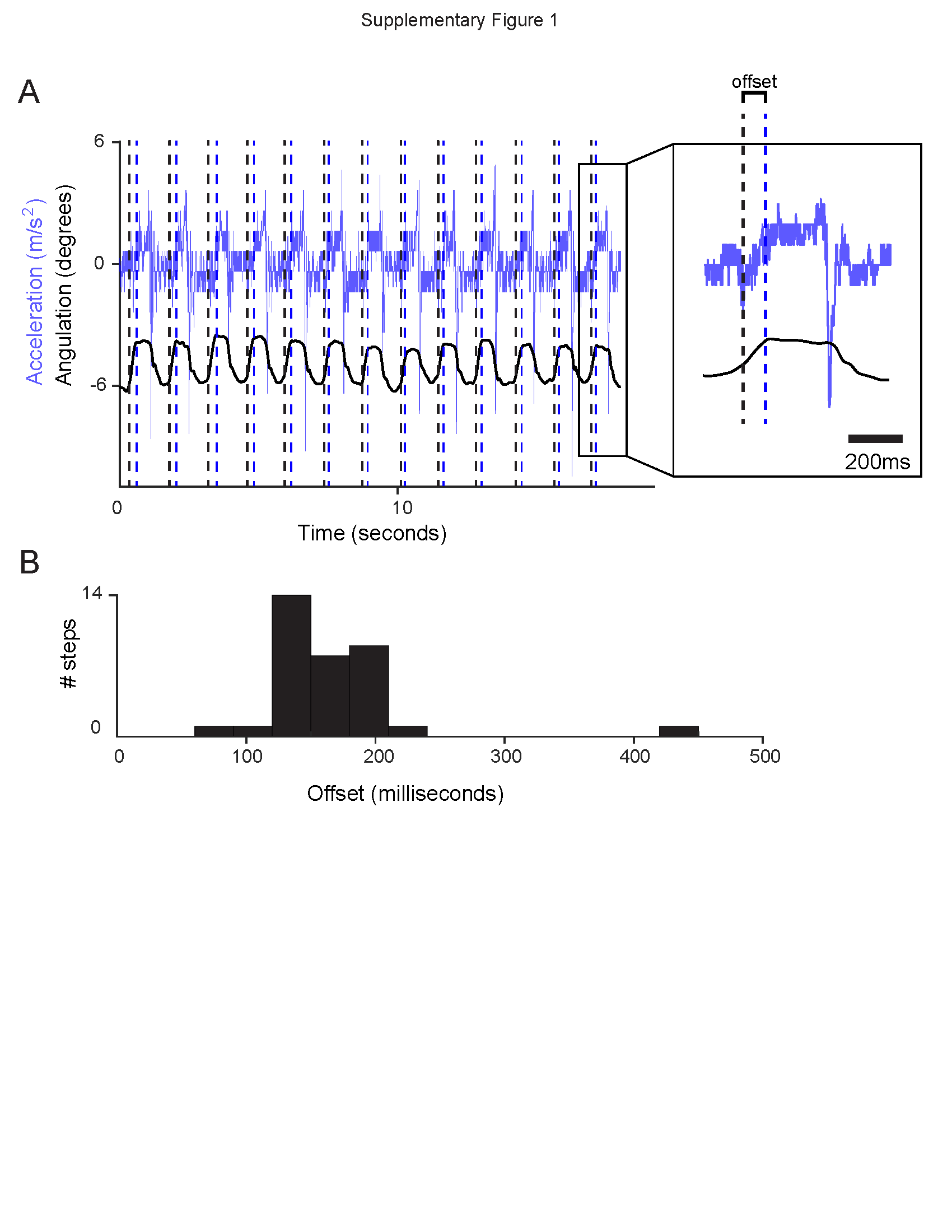

Supplement: Supplementary Figure 1 — Analysis of offset between step detection by accelerometry and step detection by inertia sensor in PD05. (A) Trace of ankle acceleration and knee angle in PD05, demonstrating noisier trace for accelerometer measurements but consistent timing and offset of acceleration trace and inertia sensor trace. (B) Histogram of offset between the two methods of detection. M = 179 ms ± SD = 97.7 ms. [file Image_1.TIF]

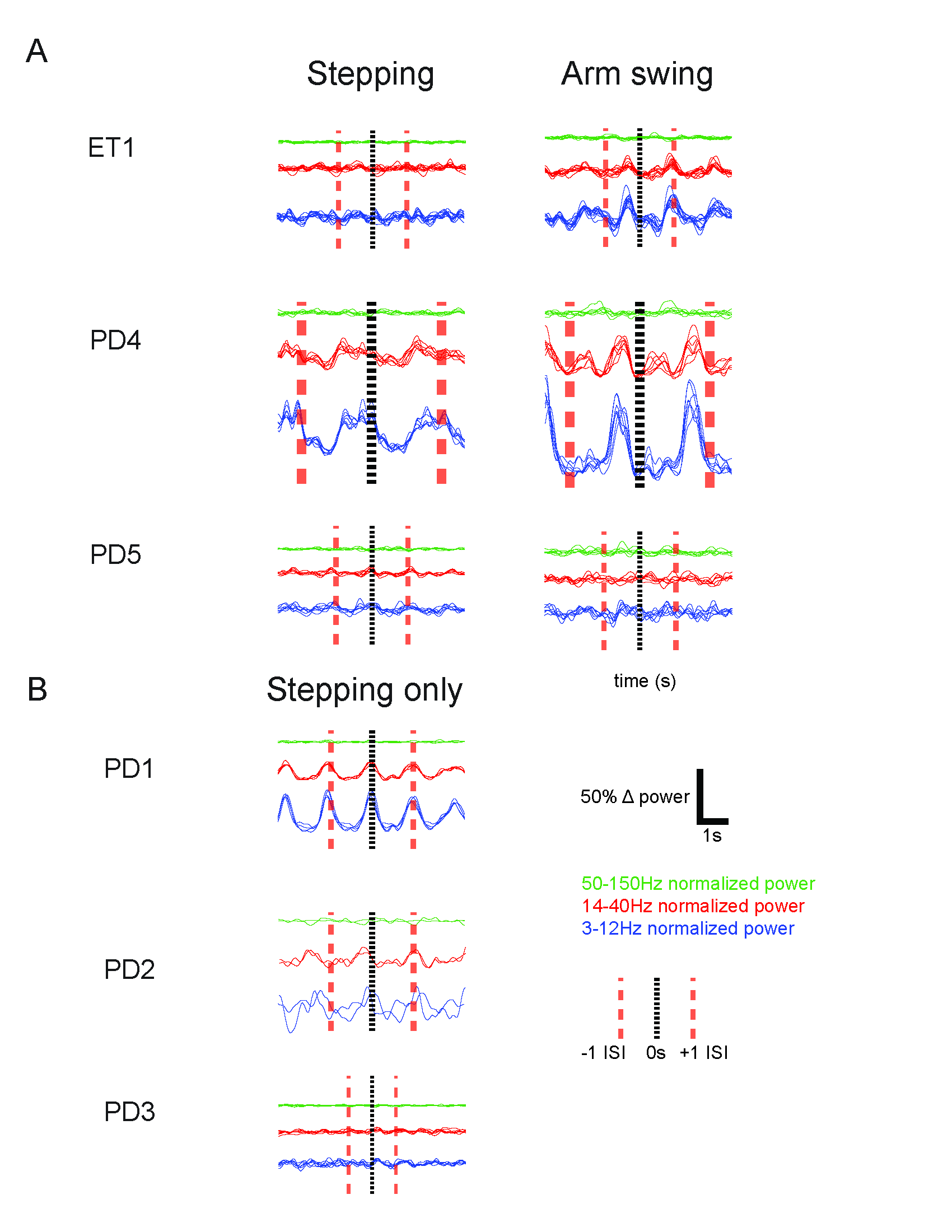

Supplement: Supplementary Figure 2 — Traces for all motor cortical contacts illustrating normalized power during stepping and arm swing for all subjects. (A) Traces of normalized power in 3–12 Hz, 14–40 Hz, and 50–150 Hz frequency ranges for subjects in which both arm swing and stepping were recorded. Each trace is one contact. (B) Traces of normalized power in the same frequency ranges for subjects in which stepping only was analyzed. Each trace is one contact. [file Image_2.TIF]

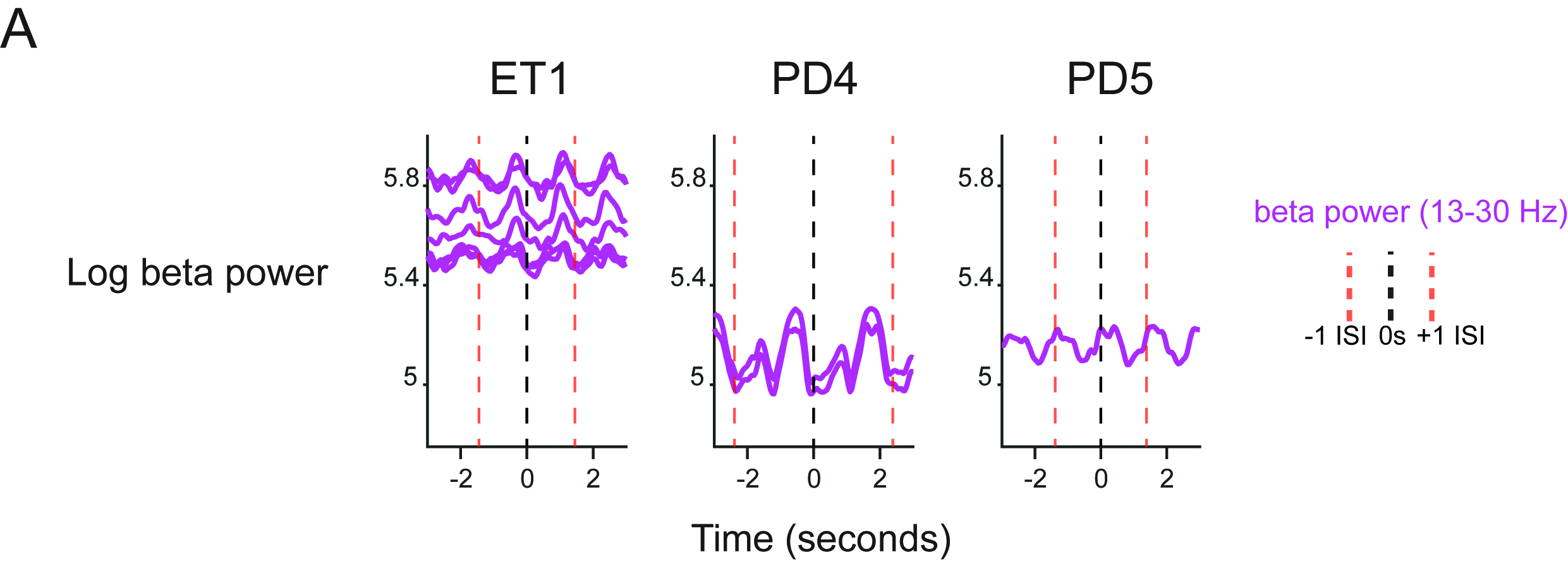

Supplement: Supplementary Figure 3 — Traces for all motor cortical contacts demonstrating significant beta modulation with movement initiation during arm swing. [file Image_3.TIF]
